# High-Quality Genome Sequences of Two Octocoral-Associated Bacteria, Endozoicomonas euniceicola EF212^T^ and Endozoicomonas gorgoniicola PS125^T^

**DOI:** 10.1128/mra.00877-22

**Published:** 2022-12-21

**Authors:** Yu-Jing Chiou, Yu-Hsiang Chen, Pei-Wen Chiang, Hsing-Ju Chen, Sen-Lin Tang

**Affiliations:** a Biodiversity Research Center, Academia Sinica, Taipei, Taiwan; b Institute of Oceanography, National Taiwan University, Taipei, Taiwan; University of Southern California

## Abstract

Endozoicomonas euniceicola EF212^T^ and Endozoicomonas gorgoniicola PS125^T^ were isolated from soft corals (Eunicea fusca and *Plexaura* sp., respectively) and sequenced using a PacBio Sequel IIe sequencer. This is the first report of the genome sequences of culturable octocoral-isolated *Endozoicomonas* strains.

## ANNOUNCEMENT

*Endozoicomonas* species (family *Hahellaceae*) are marine animal-associated bacteria (1–9). The presence of *Endozoicomonas* species is thought to be positively correlated with healthy coral conditions (10–12); however, the genome sequences of culturable octocoral-isolated *Endozoicomonas* strains remain unknown. Here, we sequenced the genomes of two culturable *Endozoicomonas* strains that were isolated from octocorals.

Endozoicomonas euniceicola EF212^T^ (NCCB 100438) and Endozoicomonas gorgoniicola PS125^T^ (NCCB 100458) were isolated from the soft corals Eunicea fusca and *Plexaura* sp., from the coasts of Florida, USA, and Bimini, Bahamas, respectively (4). Bacterial stocks were obtained from the Westerdijk Fungal Biodiversity Institute (Utrecht, Netherlands) and were cultured in MMB medium (13) at 25°C, with agitation at 200 rpm. To verify the identity of the bacterial strains, nearly full-length sequences of the bacterial 16S rRNA gene were amplified by PCR using a pair of universal primers (27F and 1541R) (14). The amplicons were checked using 1.5% agarose gel electrophoresis and purified for Sanger sequencing (3730 DNA analyzer; Thermo Fisher Scientific) by Genomics BioSci & Tech Co. (Taipei, Taiwan). The sequences for both strains corresponded to the specific 16S rRNA partial sequences in the NCBI database (GenBank accession numbers NR_109684 and NR_109685) with 100% similarity. A larger volume of bacteria was then grown for total genomic DNA (gDNA) isolation using the cetyltrimethylammonium bromide method (15). The quality and quantity of the gDNAs were checked using a NanoDrop 1000 spectrometer (Thermo Fisher Scientific) and a Qubit double-stranded DNA (dsDNA) high-sensitivity (HS) assay kit (Thermo Fisher Scientific), respectively. The gDNAs were sheared into smaller fragments, approximately 10 kb in size. The standard procedure was followed for the subsequent steps of multiplexed microbial library preparation using the SMRTbell Express template preparation kit v2.0 (Pacific Biosciences [PacBio], USA) for sequencing. Whole-genome sequencing was performed by Blossom Biotechnologies Inc. (Taipei, Taiwan) using a PacBio Sequel IIe sequencer.

**FIG 1:**
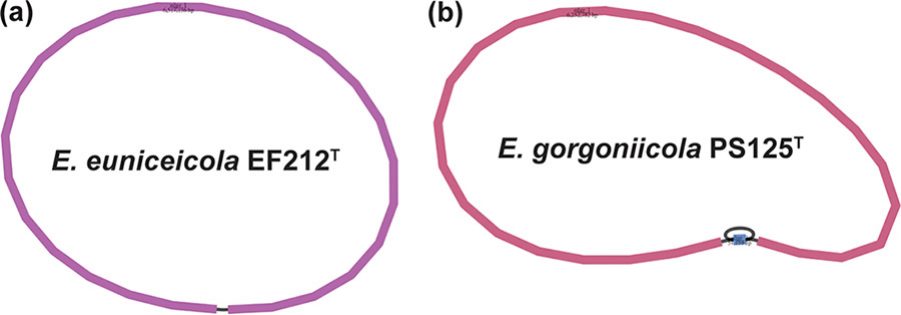
Visualization of the two assemblies using Bandage v0.9.0. (a) E. euniceicola EF212^T^. (b) E. gorgoniicola PS125^T^.

We obtained 1,415,918,326 bp raw reads (*N*_50_, 11,052 bp) for E. euniceicola EF212^T^, with a mean length of 9,987 bp, and 1,053,532,756 bp raw reads (*N*_50_, 10,207 bp) for E. gorgoniicola 1PS125^T^, with a mean length of 8,967 bp, using single-molecule real-time (SMRT) Link v10.2.1 (PacBio, USA). The raw reads were *de novo* assembled using Flye v2.9 (16), with genome coverage of 217× for E. euniceicola EF212^T^ and 166× for E. gorgoniicola PS125^T^. One contig (6,517,136 bp) of E. euniceicola EF212^T^ and two contigs (6,338,248 bp) of E. gorgoniicola PS125^T^ had the same GC content of 48%. The assembled genomes were checked for completeness, contamination, and strain heterogeneity using CheckM v1.0.18 (17) and visualized using Bandage v0.9.0 (18) (Fig. 1). The NCBI Prokaryotic Genome Annotation Pipeline (PGAP) v6.1 was used for annotation (19) (Table.1).

**TABLE 1:** Summary information for the assemblies

Parameter	Data for:	
	E. euniceicola EF212^T^	E. gorgoniicola PS125^T^
No. of reads	1,415,918,326	1,053,532,756
Raw read *N*_50_ (bp)	11,052	10,207
Genome size (Mb)	6.517136	6.338248
No. of contigs	1	2
Circular	Yes	No
GC content (%)	48	48
No. of genes	5,364	5,730
No. of coding sequences	5,229	5,598
No. of tRNAs	105	102
No. of rRNAs (5S, 16S, 23S)	25 (9, 8, 8)	25 (9, 8, 8)
Completeness (%)	98.71	99.18
Contamination (%)	2.19	1.81
Strain heterogeneity (%)	0.00	0.00
GenBank accession no.	CP103300	JAPFCC000000000

Similarity between the two strains was identified using average nucleotide identity (ANI) and average amino acid identity (AAI) calculators (20). The two strains shared high similarity (ANI, 90.0%; AAI, 86.8%), and both were close to E. montiporae CL-33^T^ (ANI, >81.6%; AAI, 79.7%), thus placing these two strains within the genus *Endozoicomonas*. Default parameters were used for all software mentioned above. According to the visualization of the assemblies using Bandage and the completeness checked using CheckM, the two genomes were determined to be a draft genome for E. gorgoniicola PS125^T^ and a complete genome for E. euniceicola EF212^T^ (Table 1 and Fig. 1).

### Data availability.

The assemblies of E. euniceicola EF212^T^ and E. gorgoniicola PS125^T^ are available under the BioProject accession number PRJNA871056. The GenBank accession numbers for E. euniceicola EF212^T^ and E. gorgoniicola PS125^T^ are CP103300 and JAPFCC000000000, respectively; the SRA accession numbers are SRX17147022 and SRX17147023, respectively.
